# From the Classroom to the Lab: How Faculty Can Extend Curriculum Oriented Research Experiences to Publish With Undergraduates

**DOI:** 10.3389/fpsyg.2019.00622

**Published:** 2019-03-27

**Authors:** Saaid A. Mendoza, Lauren E. Martone

**Affiliations:** ^1^Providence College, Providence, RI, United States; ^2^Department of Psychology, Minnesota State University, Mankato, MN, United States

**Keywords:** publishing, undergraduate research, faculty mentoring, teaching institutions, psychology curricula, liberal arts education

Faculty members who teach at liberal arts colleges or regional master's universities may face unique challenges to conducting and publishing meaningful research. Compared with doctorate-granting R1 or R2 institutions, these places of higher learning often require heavier teaching loads (e.g., 3–3 or 4–4) and provide fewer research resources, such as access to graduate students, sizable lab spaces and start-up funds, and large subject pools. Yet, we believe that these potential obstacles can be overcome by having an undergraduate curriculum that cultivates foundational research skills and committed faculty mentorship that can further develop these proficiencies through immersive and collaborative lab groups. Here, we take a dual-perspective approach by first summarizing the overarching goals of these pedagogical practices (SM), and then providing a former student's (LM) insight on the academic impact of such research experiences.

## Curriculum Oriented Research Experiences (SM)

Providence College is a private liberal arts institution with ~4,300 undergraduate and 600 graduate students. Like other colleges that offer some master's and no doctoral programs, it relies on an intimate learning environment to prepare its student body for future success. Indeed, the small student-faculty ratios typically found at these schools can result in more hands-on and personalized scientific training than at large research universities (Cech, 1999; Kaiser et al., 2014). Providing undergraduates with immersive research experiences is particularly critical, given their established benefits to student engagement, intellectual achievement, and post-graduate opportunities (Elmes, 2002).

Our psychology department offers a 12-course major that exposes students to a wide variety of subdisciplines that are grouped in domains and offers them specialized seminars to go further in depth in their preferred areas. Throughout these courses, there is a strong emphasis on the scientific nature of the field and the development of critical thinking and writing skills via curriculum oriented research experiences (henceforth referred to as C.O.R.E. strictly for the purpose of this article). During the first 2 years of the major, our declared students take a year-long sequence of Research Design and Statistical Analysis (RDSA). Rather than splitting the topics into separate semesters, we find that this integrative approach better synthesizes the theoretical and practical aspects of research, thereby making the material more accessible to students (see Barron and Apple, 2014). Within the lecture-based portion of the class, students learn about ethical responsibilities, sampling techniques, measurement, and study design, as well as practice reading, summarizing, and critiquing research. Within the complementary weekly lab, students work in groups of 2–4 to design and conduct consecutive research studies, usually a survey-based project followed by an experiment. Selection of research topics, creation of study materials, and data collection are supervised by the instructor, but are primarily completed by the students. Over the course of the year, they also practice data entry with SPSS and learn to perform reliability, correlational, regression, *t*-test, and ANOVA analyses. Students culminate their projects by individually writing up their results in an APA-style manuscript and presenting a formal poster in a campus-wide psychology conference organized by our Psi Chi chapter.

By getting involved in every step of the research process early on, our majors are able to form the analytical skills necessary for them to succeed in more concentrated research experiences (see Perlman and McCann, 2005). By their junior year, students can begin to take an advanced lab in their preferred sub-discipline, such as Animal Learning, Bio, Cognitive, Developmental, Health, Neuro, and Social Psychology. These small seminars center around the discussion of empirical articles and have a lab component in which students develop more complex research studies that are presented as talks in our local psychology conference. Although certain students may be ready to join and contribute to a faculty lab earlier in their studies, we believe that the step-wise progression of the C.O.R.E. helps develop our majors into critical consumers and producers of research, and better prepares them to collaboratively conduct publishable research.

## Impact: A Student Perspective of C.O.R.E. (LM)

The general consensus in the literature is that smaller classroom sizes promote student engagement, commitment, and motivation (Mulryan-Kyne, 2010). Further, students who perceive faculty members to be approachable and available tend to report greater intrinsic motivation and academic achievement (Komarraju et al., 2010). These components were indeed reflected in my educational experience at Providence College. Right from the start of my psychology major, an optimal learning dynamic was achieved by faculty members who were able to fully draw in their students by making the course content more personally relevant. This individualized classroom experience was especially critical to engaging me in research. As a sophomore taking RDSA, the intimate classroom setting and increased faculty support motivated me to succeed in a course that can produce anxiety in many students (Sandoz et al., 2017). Statistics and research design can often feel dull or difficult for students to whom these concepts do not yet have pertinence. However, independently designing and conducting research projects lessened these feelings for me and drove my desire to thoroughly learn the challenging material (Kember et al., 2008).

Without students gaining a solid foundation in statistics and research design, their lab contributions would be limited in a manner that could directly affect faculty productivity. The time and effort required to mentor students, as well as the lack of foundational skills obtained by the students, can serve as roadblocks to producing meaningful research collaborations between professors and undergraduates (Johnson et al., 2015; Brew and Mantai, 2017). My education at Providence College minimized these obstacles by emphasizing the empirical nature of psychology and equipping me with adequate research skills by the time I joined a faculty member's lab during my junior year. Consequently, I was able to focus my lab efforts on refining these proficiencies and developing my individual research identity, both of which prepared me for graduate school.

## Faculty Development & Mentorship in Research Labs (SM)

Although critical to the training of undergraduates, a research-focused curriculum should be seen as only the first step toward productive collaborations between students and faculty. To further promote research success, professors should consider using a graduate school model to select, develop, and mentor their lab members. For instance, our Social Perception and Attitudes Lab requires all interested individuals to submit a written application, along with a copy of their résumé and academic transcript. Students who have completed the preferred course pre-requisites and thoughtfully expressed their research interests and intentions are then invited for a personal interview. This extensive process generally produces applicants who are truly motivated to engage in the research process and pursue the principle investigator's questions. Furthermore, since new members usually serve as volunteers before being offered course credit, the lab is comprised of undergraduates who often display a high level of intrinsic motivation (Deci, 1971).

In line with goal setting theory (Locke and Latham, 2002), lab members fill out learning agreements each semester in which they identify specific and challenging (yet reachable) goals. These “educational contracts” are discussed in individual meetings throughout the semester and serve the dual purpose of motivating students and holding professors accountable. Having already received initial training through their course curriculum, undergraduates are able to take a more active role in the research process. Students work closely with the principle investigator to identify gaps in the literature, create study materials, write IRB protocols, program studies, run subjects, and participate in data analysis. Instead of being trained on the basics of research, undergraduates focus their lab contributions on formulating theoretically grounded hypotheses and developing impactful studies that are more likely to be published.

Faculty members who seek productive collaborations with undergraduates must also be willing to serve as mentors. This begins with instilling confidence in the students, both on an interpersonal and academic level. One effective way to accomplish this is by having research assistants work in pairs on projects of primary interest to them. Teamwork not only increases the lab's output, but it also helps students dive deeper into specific research questions and build their expertise (Waite and Davis, 2006a). Through this process, students become increasingly comfortable with discussing research findings and ultimately presenting them at conferences. Such opportunities are invaluable, as students are exposed to a variety of psychological disciplines and are able to begin networking with the aid of their faculty mentor. Moreover, the constructive feedback provided during poster sessions can inspire students to independently pursue new research questions and add to their overall professional development (Thiry et al., 2011). Students at this stage should be encouraged to apply for undergraduate research grants, which help produce publishable projects with larger, more variable samples that can be obtained through paid platforms like Amazon MTurk. Lastly, when lab members fulfill the expectations for authorship set forth by the American Psychological Association, faculty members should invite their students to draft sections of the manuscript, making sure to also include them in the submission and review process so they can become familiar with these crucial aspects of research. It is important to be open-minded about possible publication outlets, as some journals often have certain sections dedicated to single-study projects that can be feasibly carried out and co-authored by undergraduates who are working with a condensed timeline. In short, taking on the full responsibility of a mentor can help professors at teaching institutions produce fruitful collaborations with their undergraduates and send students down the right path early in their research careers.

## Impact: A Student Experience in a Faculty Lab (LM)

While the curriculum at Providence College served as a catalyst for my interest in research, working collaboratively with a faculty member and like-minded peers fully engaged me in the scientific process. First, my faculty advisor and I filled out a learning contract to agree on expectations and goals for the semester. This helped instill a sense of assurance that the faculty member would provide practical and personal support, and that I as a student would provide my active effort and dedication to the lab. Further, students work in pairs on projects of mutual interest. Collectively making decisions, discussing ideas, and trouble-shooting problems heightened our individual motivation to carry out quality research (Waite and Davis, 2006b). Moreover, joining a faculty-led lab allowed me to move beyond the C.O.R.E. and elevated my critical thinking skills to a level appropriate for graduate school. By actively taking part in every aspect of the research process as an undergraduate, I was a stronger candidate for graduate school (Karazsia and Smith, 2016), and once admitted, was able to immediately begin making progress toward my independent master's projects and thesis proposal.

I attribute most of my preparedness to the opportunities provided by my faculty mentor. He served as a model for how to formulate theory-based research questions, a teacher for how to carry out publishable research, and a guide for choosing paths that would lead to professional success, even as an undergraduate. Combined with my dedication to research, these mentorship qualities led me to presenting my lab project as a first-author poster at the Association for Psychological Science Annual Convention. More importantly, I was able to work with my mentor during my senior year on a special issue article submission that was accepted for publication. The feedback and multi-disciplinary exposure during both of these experiences expanded my knowledge of psychological research, enhanced my writing skills, and increased my self-confidence as a scientist (Helm and Bailey, 2013). Opportunities such as these promote the production of meaningful publishable research as an undergraduate, and especially now as a graduate student.

## Conclusion

In order for psychological research to progress, it is necessary for scientists to be fully committed to teaching and mentoring students, beginning at the undergraduate level. By offering a curriculum that emphasizes the scientific nature of psychology, departments can develop students into confident and competent researchers who are then able to collaborate with invested faculty members. Although this dual-prong approach does not necessarily guarantee publication success, we believe that it can serve as a recipe for increasing faculty and student productivity at more teaching-focused institutions that have limited research resources.

## Author Contributions

SM drafted the outline of the paper with the help of LM. Both SM and LM contributed to the writing and editing of the article, with SM taking responsibility for its submission and proofing.

### Conflict of Interest Statement

The authors declare that the research was conducted in the absence of any commercial or financial relationships that could be construed as a potential conflict of interest.
